# Vav3-Deficient Astrocytes Enhance the Dendritic Development of Hippocampal Neurons in an Indirect Co-culture System

**DOI:** 10.3389/fncel.2021.817277

**Published:** 2022-02-14

**Authors:** David Wegrzyn, Josephine Zokol, Andreas Faissner

**Affiliations:** Department of Cell Morphology and Molecular Neurobiology, Ruhr-University Bochum, Bochum, Germany

**Keywords:** Vav3, Rho-GTPases, guanine nucleotide exchange factor, astrocytes, hippocampal neurons, neuron-astrocyte interaction, cytokines, CCL5

## Abstract

Vav proteins belong to the class of guanine nucleotide exchange factors (GEFs) that catalyze the exchange of guanosine diphosphate (GDP) by guanosine triphosphate (GTP) on their target proteins. Here, especially the members of the small GTPase family, Ras homolog family member A (RhoA), Ras-related C3 botulinum toxin substrate 1 (Rac1) and cell division control protein 42 homolog (Cdc42) can be brought into an activated state by the catalytic activity of Vav-GEFs. In the central nervous system (CNS) of rodents Vav3 shows the strongest expression pattern in comparison to Vav2 and Vav1, which is restricted to the hematopoietic system. Several studies revealed an important role of Vav3 for the elongation and branching of neurites. However, little is known about the function of Vav3 for other cell types of the CNS, like astrocytes. Therefore, the following study analyzed the effects of a *Vav3* knockout on several astrocytic parameters as well as the influence of Vav3-deficient astrocytes on the dendritic development of cultured neurons. For this purpose, an indirect co-culture system of native hippocampal neurons and Vav3-deficient cortical astrocytes was used. Interestingly, neurons cultured in an indirect contact with Vav3-deficient astrocytes showed a significant increase in the dendritic complexity and length after 12 and 17 days *in vitro* (DIV). Furthermore, Vav3-deficient astrocytes showed an enhanced regeneration in the scratch wound heal assay as well as an altered profile of released cytokines with a complete lack of CXCL11, reduced levels of IL-6 and an increased release of CCL5. Based on these observations, we suppose that Vav3 plays an important role for the development of dendrites by regulating the expression and the release of neurotrophic factors and cytokines in astrocytes.

## Introduction

Vav3 belongs to the Dbl-family of guanine nucleotide exchange factors (GEFs) which includes further 68 members in humans (Rossman et al., 2005). It is characterized by the Dbl homology (DH) domain localized next to a regulatory pleckstrin homology (PH) domain and can be activated by the phosphorylation of a tyrosine residue (Tyr174) (Turner and Billadeau, 2002; Rossman et al., 2005). The catalytic activity of Vav-GEFs is accomplished by an interaction between the DH domain and target proteins. Here, especially members of the Rho-GTPase family can be switched into an active state by Vav-GEFs. Vav3 can directly activate RhoA, RhoG and Rac-1 (Movilla and Bustelo, 1999; Movilla et al., 2001). Furthermore, a study described that Vav3 indirectly acts on Cdc42 by an accumulation of phosphatidylinositol 3,4,5- trisphosphate (PIP_3_) (Aoki et al., 2005). Since RhoA, Rac-1 and Cdc42 are strong modulators of the cytoskeleton, Vav3 functions as a mediator of intracellular reorganizations of the cytoskeleton (Tapon and Hall, 1997). In this way, Vav3 regulates key events of CNS development like migration (Khodosevich et al., 2009), proliferation (Fujikawa et al., 2002), differentiation (Ulc et al., 2019) and axo-dendritic maturation (Quevedo et al., 2010; Sauzeau et al., 2010; Ulc et al., 2017; Wegrzyn et al., 2020).

Interestingly, several studies could show that a lack of Vav3 impacts the axonal and dendritic morphology of neurons in different brain areas. Purkinje and granule cells of *Vav3*-knockout animals showed a reduction of the dendritic complexity in the postnatal period of mice (Quevedo et al., 2010). This observation was accompanied by motoric deficiencies which, however, vanished in the adult stage. Furthermore, GABAergic neurons exhibited an impaired axon guidance in the brainstem when Vav3 was deficient (Sauzeau et al., 2010). The altered axon guidance of GABAergic neurons in the brainstem of mice was consequently linked to hypertension, tachycardia, and renocardiovascular dysfunctions (Sauzeau et al., 2010). Additionally, primary hippocampal neurons derived from Vav3-deficient embryonic animals displayed a significant raise in their axonal lengths and complexities as well as in their structural synapse numbers *in vitro* (Wegrzyn et al., 2020). In schizophrenia patients *VAV3* could be furthermore identified as a risk gene and polymorphisms were discussed to possibly be responsible for reduced volumes of the left superior and middle temporal *gyri* (Aleksic et al., 2013). Although, there are several studies with a focus of Vav3 on the neuronal development and differentiation, less is known about the function of Vav3 in astrocytes. Astrocytes are glial cells that highly support neurons in multiple ways (Ben Haim and Rowitch, 2017). With their protrusions, astrocytes form perisynaptic contacts and contribute to the tripartite synapse (Perea et al., 2009; Faissner et al., 2010; Bernardinelli et al., 2014). Furthermore, astrocytes are involved in the uptake and recycling of glutamate (Norenberg and Martinez-Hernandez, 1979), modulation of neuronal activity by the release of gliotransmitters (Zhang et al., 2003; Panatier et al., 2006; Jourdain et al., 2007; Henneberger et al., 2010), structure of the blood-brain barrier (Janzer and Raff, 1987) and metabolic support of neurons (Deitmer et al., 2019). There is a growing evidence that Rho-GTPases affect the stellation of astrocytes and modify neuron-astrocyte interactions (Zeug et al., 2018). Furthermore, different roles of RhoA, Rac-1 and Cdc42 could be observed for the morphological organization of astrocytes. RhoA has been identified to mainly act as a negative regulator of the astrocytic process outgrowth. The inhibition of RhoA by C3 derived from *Clostridium botulinum* enhanced the outgrowth and branching of astrocytic processes as well as the migratory activity in an *in vitro* scratch wound assay (Höltje et al., 2005). Similar results were obtained when the RhoA downstream target protein Rho-associated protein kinase (ROCK) was specifically inhibited via the selective inhibitor Y27632 (Höltje et al., 2005). Contrary effects could be seen for Rac-1. The transfection of primary astrocytes with Rac1-shRNA disrupted the formation of a stellate morphology when Y27632 was added to the culture medium (Racchetti et al., 2012). Additionally, studies with a dominant negative form of Rac1 could support this observation and furthermore show a reduced motility of astrocytic processes which went along with a reduced maturation of dendritic protrusions in organotypic slice cultures (Nishida and Okabe, 2007). The astrocytic migration is furthermore positively promoted by the activity of Rac1 (Liao et al., 2015). For Cdc42, especially an important role for the migration could be observed (Robel et al., 2011; Bardehle et al., 2013).

While there are several scientific publications about the individual impact of single Rho-GTPases on astrocytic properties, less is known about the role of regulatory upstream GEFs like Vav3. Here, we utilized an indirect co-culture system to investigate the impact of a *Vav3* knockout in astrocytes on the dendritic morphology of primary hippocampal neurons. Furthermore, we characterized the expression levels of several neurotrophic factors on mRNA level and the cytokine levels in the cell culture supernatant of native and Vav3-deficient astrocytes. A better understanding of Rho-GTPases and their upstream regulators might unravel novel aspects of neuron-astrocyte interactions and allow for new therapeutic strategies by the manipulation of Rho-GEFs.

## Materials and Methods

### Animals

All experiments of this study were performed in accordance with the Society for Neuroscience and the European Council Directive of September 22, 2010 (2010/63/EU), for care of laboratory animals and approved by the animal care committee of North Rhine-Westphalia, Germany, based at the LANUV (Landesamt für Umweltschutz, Naturschutz und Verbraucherschutz, North Rhine-Westphalia, D-45659 Recklinghausen, Germany). Wildtype (WT) SV129 and *Vav3* knockout animals of both sexes were used and originated from the mouse colony of the Department for Cell Morphology and Molecular Neurobiology of the Ruhr-University Bochum (Luft et al., 2015; Wegrzyn et al., 2020). The animals were kept under standardized conditions with a regulated temperature (21°C), humidity (60–70%) and a 12 h dark/light circle. The animals were supplied with food and water *ad libitum*.

### Cell Culture

#### Preparation of Mixed Glial Cultures

Primary mixed glial cultures derived from cerebellar tissue of SV129 and *Vav3^–/–^* animals and were performed as previously described, with slight modifications of the protocol (McCarthy and de Vellis, 1980; Gottschling et al., 2016). First, postnatal day 1–3 (P1-3) animals were decapitated, and six cortices were transferred into 1 ml HBSS (Thermo Fisher Scientific Inc.; Cat. No.: 14170088). Afterward, the HBSS was replaced by 1 ml digestion solution composed of 30 U Papain (Worthington; Cat. No.: LS003126), 80 μg/ml (w/v) DNase I (Worthington; Cat. No.: LS002007), and 0.96 mg/ml (w/v) L-cysteine (Sigma-Aldrich; Cat. No.: C2529) in DMEM (Thermo Fisher Scientific Inc.; Cat. No.: 41966029) and incubated for 1 h at 37°C Contemporaneous, a T-75 culture flask (Sarstedt; Cat. No.: 83.3911.002) was coated with 10 μg/ml (w/v) poly-D-lysine (PDL, Sigma-Aldrich; Cat. No.: P0899) for 1 h at 37°C in the incubator. Afterward, the digestion solution was removed and replaced by freshly prepared astrocyte culture medium consisting of DMEM (Thermo Fisher Scientific Inc.; Cat. No.: 41966029) with 10% (v/v) horse serum (Sigma-Aldrich; Cat. No.: S9135) and 0.1% (v/v) gentamicin (Sigma-Aldrich; Cat. No.: S9135). After the digestion, the cortices were triturated and centrifuged at 1,000 rpm for 5 min. Next, the supernatant was discarded, and the pellet resuspended in 1 ml of astrocyte medium. Finally, the cell solution was added to 9 ml astrocyte medium in the T-75 culture flask, which was previously washed with PBS (1x) after the coating procedure. The cultures were kept at 37°C and 6% CO_2_ with a first medium exchange after 7 days and the following medium exchanges were performed every second or third day.

#### Elimination of Microglia and Oligodendrocyte Precursor Cells

In order to receive pure astrocytic cultures, the T-75 culture flasks were placed on an orbital shaker for 1 h at 250 rpm and 37°C (New Brunswick Scientific) after an initial cultivation time of 7–8 days. In a second step, the flasks were shaken at 250 rpm and 37°C overnight after the lid of culture flask was covered with parafilm (Brand, Wertheim). This procedure accomplished a constant CO_2_ level in the flasks. The following day, the culture medium was completely removed and replaced by fresh and pre-warmed astrocyte medium which additionally contained 20 mM of cytosine-1-β-D-arabinofuranoside (Ara-C, Sigma-Aldrich, Cat. No.: C1768). The treatment of the cells with Ara-C was maintained for 48–72 h and reduced the number of microglia and Oligodendrocyte Precursor Cells (OPCs). Finally, pure astrocyte cultures were kept at 37°C and 6% CO_2_ with a complete medium exchange every second or third day until further experiments were performed.

#### Scratch Wound Healing Assay

For the scratch wound assay, 4-well plates (Thermo Fisher Scientific Inc.; Cat. No.: 176740) were used which were previously coated with 10 μg/ml (w/v) PDL. Wildtype or *Vav3*^–/–^astrocytes were detached from the previously prepared T-75 flasks via an exchange of the astrocyte culture medium by 3 ml of 0.05% (v/v) trypsin/EDTA (T/E) (Thermo Fisher Scientific Inc.; Cat. No.: 25300054). The digestion solution was left on the astrocytic monolayer for 7 min at 37°C in the incubator. Then, the detached astrocytes were transferred into 7 ml of astrocyte medium and centrifuged at 1,000 rpm for 5 min. After the centrifugation, the supernatant was discarded, and the cell pellet resuspended in 1 ml astrocyte medium. A total number of 35,000 astrocytes in 500 μl culture medium (=̂ 70,000 cells/ml) was plated out per well. The astrocytes were cultured at 37°C and 6% CO_2_ with a total medium exchange every second or third day. After a cultivation time of 10 days, astrocytes were confluently grown and a scratch was carefully drawn across the cell layer with the tip of a 10 μl pipette, as previously described (Etienne-Manneville, 2006). Afterward, the wound closure was documented after 24, 48, and 72 h via phase contrast microscopy.

#### Culturing of Astrocytes in Transwells

In order to prepare different co-culture combinations, astrocytes of both conditions were plated out in transwells with a permeable membrane (pore size: 0.4 μm; Falcon by Thermo Fisher Scientific Inc.; Cat. No. 08-770). The membrane allowed for the diffusion of astrocytic secreted factors into the commonly shared medium (Geissler and Faissner, 2012). The detachment of wildtype and *Vav3*^–/–^ astrocytes was performed as previously described in chapter 2.2.3. Here, the dissociated astrocytes were plated out in a density of 25,000 cells in 500 μl astrocyte medium (=̂ 50,000 cells/ml) on PDL-coated transwells. Finally, the transwells were hang in 24-well dishes (Thermo Fisher Scientific Inc.; Cat. No.: 142475) and placed in the incubator at 37°C and 6% CO_2_. After a cultivation time of approximately 4–5 days the transwells were used for the co-cultures.

#### Primary Hippocampal Neuronal Cultures

For the culturing of pure primary neurons, embryonic wildtype and *Vav3*^–/–^ animals of the developmental stage E15.5 were used and the embryonic hippocampi dissected, as previously described (Gottschling et al., 2016). The embryonic day 15.5 was determined for the preparation in order to obtain pure neuronal cultures without astrocytes which begin to form at earliest at E17.5 and especially in the first postnatal week (Bond et al., 2020). The use of serum-free medium prevented the proliferation of any astrocyte progenitors. Isolated hippocampi were transferred and collected in dissection medium consisting of HBSS (Thermo Fisher Scientific Inc.; Cat. No.: 14170088), 0.6% (w/v) glucose (Serva Electrophoresis GmbH; Cat. No.: 22700), and 10 mM HEPES (Thermo Fisher Scientific Inc.; Cat. No.: 15630080). After the dissection, the tissue digestion was performed using 30 U Papain (Worthington; Cat. No.: LS003126) in MEM (Thermo Fisher Scientific Inc.; Cat. No.: 31095029) with 80 μg/ml (w/v) DNase I (Worthington; Cat. No.: LS002007) and 0.96 mg/ml (w/v) L-cysteine (Sigma-Aldrich; Cat. No.: C2529) for 15 min at 37°C. Next, the digestion solution was aspirated and replaced by fresh neuron medium composed of MEM (Thermo Fisher Scientific Inc.; Cat. No.: 31095029), 2% (v/v) B27 (Thermo Fisher Scientific Inc.; Cat. No.: 17504044), 0.1% (v/v) ovalbumin (Sigma-Aldrich; Cat. No.: A7641) 10 mM sodium pyruvate (Sigma-Aldrich; Cat. No.: S8636), and 0.1% (v/v) gentamicin (Sigma-Aldrich; Cat. No.: S9135). The hippocampi were washed three times by exchanging the neuron medium. After the final washing step, the tissue was carefully triturated to a single cell suspension which was subsequently used for the co-cultures.

#### Indirect Co-culturing of Neurons and Astrocytes

Primary hippocampal neurons were cultured on poly-L-ornithine (PLO) (15 μg/ml; Sigma-Aldrich; Cat. No.: P3655) coated glass cover slips in special 24-well plates which were suitable for the astrocyte containing transwells (Falcon; Product No.: 353504). A total number of 35,000 wildtype or *Vav3*^–/–^ neurons was plated out in 500 μl neuron medium per well (=̂ 70,000 cells/ml). Afterward, the well plates were placed in the incubator for 1 h to allow the cells to attach to the cover slips. During this incubation time, the serum-containing astrocyte medium in the transwells was replaced by serum-free neuron medium. Then, the astrocyte containing transwells were carefully hung in the 24-well plates with the neurons. The co-cultures were kept in the incubator at 37°C and 6% CO_2_ until further experiments were performed. After a cultivation time of 12 and 17 days *in vitro*, the transwells were removed and the neuronal networks were fixed with pre-warmed 4% (w/v) paraformaldehyde (Carl Roth GmbH & Co., KG; Cat. No.:4235.1) for 10 min. After three washing steps with PBS, the cells were kept at 4°C until the immunocytochemical visualization of the dendrites was performed.

### Immunocytochemistry

#### Visualization of Dendritic Trees

After the fixation, neurons were incubated with primary antibodies diluted in PBT-1 (PBS with 0.1% v/v Triton X-100 (AppliChem GmbH; Cat. No.: A4975,0500; Sigma-Aldrich) and 1% w/v bovine serum albumin (BSA) (Carl Roth GmbH & Co., KG; Cat. No.:8076.2). Here, the following antibodies were used: anti-Map2 [1:300, rabbit, polyclonal, RRID: AB_1840999; Sigma-Aldrich (by Merck KGaA)], anti GAD65/76 antibody (1:300, mouse, monoclonal, RRID: AB_2039129, Enzo Life Sciences). Since interneurons form morphologically different dendritic trees compared to pyramidal neurons, GAD65/67 was used to distinguish between these two neuronal types. The incubation was carried out in a humid chamber for 1 h at room temperature. Then, the antibody solution was aspirated, and the coverslips were washed three times with PBS/A (PBS with 0.1% (w/v) BSA). Then, the cover slips were incubated with the secondary antibody solution containing the following antibodies: anti-rabbit (Cy3, polyclonal, goat, RRID: AB_2338003, Jackson ImmunoResearch Labs), anti-mouse (AF488, polyclonal, goat, RRID:AB_AB_2338844, Jackson ImmunoResearch Labs). Furthermore, bisbenzimide (Hoechst 33258, 1:100.000, Sigma-Aldrich) was used in a dilution of 1:1,00,000 in the secondary antibody solution for the visualization of nuclei. The secondary antibodies were left for 45–60 min on the coverslips at room temperature. Afterward, the coverslips were rinsed three times with PBS (1x) and once with purified Milli-Q water. In a final step, the coverslips were placed on microscope slides (Thermo Fisher Scientific Inc.; Cat. No.: 630-2012) and mounted with ImmuMount (Thermo Fisher Scientific Inc.; Cat. No.: 10662815).

### Molecular Biology

#### Sample Collection and mRNA Isolation

Previously prepared wildtype and *Vav3*^–/–^ astrocytes were detached from the T-75 flasks as described in chapter 2.2.3. Then, a total number of 200,000 astrocytes was plated out in 3 ml astrocyte medium (=̂ 66,667 cells/ml) per well. Here, PDL-coated 6-well plates (Thermo Fisher Scientific Inc.; Cat. No.:140675) were used. After a cultivation time of 10 days *in vitro*, the cells were lysed following the manufacturers instruction. The GenElute Mammalian Total RNA Miniprep Kit (Sigma-Aldrich by Merck KGaA; Cat. No.: RTN350) was used for the isolation and purification of the mRNA. Furthermore, the lysates of two wells were pooled in order to obtain a sufficient amount of mRNA for further analysis.

#### cDNA Synthesis

The cDNA synthesis was performed with the First Strand cDNA Synthesis Kit from Thermo Fisher Scientific Inc. (Cat. No.: K1622). Here, 1 μg mRNA was utilized for the synthesis of cDNA which was subsequently stored at –20°C until the Polymerase chain reactions (PCR) were performed.

#### Reverse Transcriptase-PCR

The reverse transcriptase (RT) PCRs were performed with the primer pairs listed in Table 1. All primers were ordered and obtained from Sigma-Aldrich.

**TABLE 1:** Primer sequences and PCR settings.

Gene	Primer sequence	Product length	Cycle number	Annealing temperature
*Tsp1* for	5′ GCTGCCAATCATAACCAGCG 3′	415 bp	30	64°C
*Tsp1* rev	5′ GGTTGTTTGGCGGTGAGTTC 3′			
*Tsp2* for	5′ AGAGAGAGCCAGTCCGATGT 3′	732 bp	30	63°C
*Tsp2* rev	5′ TCGATAAGATCGCAGCCCAC 3′			
*NT-3* for	5′ ATCCAAGGCAACAGCATGGA 3′	474 bp	30	64°C
*NT-3* rev	5′ CTGGTGTCCCCGAATGTCAA 3′			
*NGFv1* for	5′ GGGAGCGCATCGAGTGAC 3′	264 bp	30	62°C
*NGFv1* rev	5′ CCTCACTGCGGCCAGTATAG 3′			
*NGFv2* for	5′ GGGAGCGCATCGAGTTTT 3′	341 bp	30	63°C
*NGFv2* rev	5′ CTGTCACTCGGGCAGCTATT 3′			
*TGF-*β for	5′ ATGCTAAAGAGGTCACCCGC 3′	452 bp	30	64°C
*TGF-*β rev	5′ GTTCATGTCATGGATGGTGCC 3′			
*BDNF* for	5′ GACGACATCACTGGCTGACA 3′	372 bp	32	64°C
*BDNF* rev	5′ TGCTTCAGTTGGCCTTTGGA 3′			
*CNTF* for	5′ TTAGGGGATGGCTTTCGCAG 3′	317 bp	30	66°C
*CNTF* rev	5′ CACCTTCAGTCGGGGTGAAA 3′			
*LIF* for	5′ GAGAGCTAAGGGGACTGGGA 3′	559 bp	30	62°C
*LIF* rev	5′ AAGTACCTTTGCGACCTCCG 3′			
*Vav3 for*	5′ CAGTGGCTCATCCACAGCA 3′	237 bp	30	62°C
*Vav3 rev*	5′ TCCAAAGGIATCGCAGCAGG 3′			
*Actin* for	5′ TATGCCAACACAGTGCTGTCTGG 3′	247 bp	30	62°C
*Actin* rev	5′ AGAAGCACTTGCGGTGCACGATG 3′			

### Medium Conditioning and Cytokine Array

For the analysis of the secreted cytokines in the supernatant of wildtype and *Vav3*^–/–^ astrocytes, the Proteome Profiler Mouse Cytokine Array Panel A (R&D Systems, Cat. No.: ARY006) was used without changing the manufacturers protocol. Here, the astrocytes were cultured in T-25 flasks (Sarstedt; Cat. No.: 83.3911.002) which were previously coated with 10 μg/ml (w/v) PDL. After purifying the cultures from microglia and OPCs (10–11 days in culture) the astrocyte medium was replaced by serum-free neuron medium composed of MEM (Thermo Fisher Scientific Inc.; Cat. No.: 31095029), 2% (v/v) B27 (Thermo Fisher Scientific Inc.; Cat. No.: 17504044), 0.1% (v/v) ovalbumin (Sigma-Aldrich; Cat. No.: A7641) 10 mM sodium pyruvate (Sigma-Aldrich; Cat. No.: S8636), and 0.1% (v/v) gentamicin (Sigma-Aldrich; Cat. No.: S9135) and conditioned for 24 h. Then, the supernatant was collected and stored at –80°C until the cytokine array analysis was performed. For the cytokine array 1 ml of the astrocyte supernatant was used for every experimental repetition. The chemiluminescence signals were measured with a chemiluminescence reader from Biostep and an exposure time of 2 min was set. The quantification of the signals was performed via ImageJ and the signal intensities were normalized against the internal positive controls of the array.

### Neurotrophin-3 ELISA

For the detection of NT-3 in the supernatant of wildtype and *Vav3*^–/–^ astrocytes, serum-free neuronal medium was conditioned for 24 h, as previously mentioned under 2.5. Then, the Mouse Neurotrophin-3 ELISA Kit (ab213882) from Abcam was used without any changes of the manufacturers protocol.

### Microscopy

The immunocytochemically stained neurons were recorded with the fluorescence microscope AxioPlan 2 from Zeiss with an affiliated digital camera (AxioCam MRm, Zeiss). Furthermore, the associated AxioVision 4.5 software (Zeiss) was utilized for the data documentation. For the phase contrast images of the astrocytes and neuronal networks the light microscope AxioObserver A1 (Zeiss) was used.

### Data Quantification

The dendritic arborization was analyzed using the “Sholl analysis v3.0” plugin for ImageJ which is freely available at https://imagej.net/plugins/sholl-analysis (Sholl, 1953; Ferreira et al., 2014). Here, the immunocytochemical recordings were first transformed into binary images and then skeletonized. Afterward, a region of interest (ROI) was defined, and the radius step size was set at 15 μm. The obtained data points were transferred to Microsoft Excel for further quantification. Additionally, the dendritic lengths and the number of dendrites were manually measured with the “Freehand line” tool and the “Multi-point” tool in ImageJ (v 1.53k). The morphological analysis of this study was limited to the dendritic compartment since the axons were extensively branched and highly wired with axons of other neurons, at this culturing stage. A quantification of single axons was consequently not possible in this culture system.

For the measurement of the scratch area in the astrocytic monolayer the “Freehand Selections” tool was utilized after defining a scale bar.

The results of the RT-PCRs were quantified with the “Rectangle” tool in ImageJ. Here, the mean gray value of every PCR band was measured. After subtracting the background, the values were set in relation to the intensity of the actin bands.

The signal intensities of the cytokine array were evaluated similarly. However, the “Oval” tool in ImageJ was used instead of the “Rectangle” tool. Here, also the mean gray values were measured and set in relation to the internal positive controls after subtracting the background noise.

### Statistics

The statistical analysis of this study was carried out with GraphPad Prism (v. 8.2.1). For the Sholl analysis as well as for the dendrite lengths and branches the normality distribution of the data sets was tested with the Kolmogorov-Smirnov test. Afterward, the Kruskal-Wallis test with a Dunn’s multiple comparison test was performed to determine the significance level between the groups. For a better and clearer overview the data were shown in a pairwise manner in individual graphs. A detailed description about the number of experimental repetitions as well as the total number of analyzed neurons can be found in the figure legends.

For the scratch wound assay the normality distribution of the data sets was also tested via the Kolmogorov-Smirnov test. Here, normally distributed data sets were statistically analyzed with the unpaired *t*-test. A detailed description about the number of analyzed recordings is also given in the associated figure legends.

The normality distribution of the PCR data sets was tested with the Shapiro-Wilk-test. Since all data sets were normally distributed, the unpaired *t*-test was performed to determine the significance levels. This was also the case for the data sets from the cytokine array analysis. The associated figure legends give a detailed description about the number of experimental repetitions and replicates.

## Results

### *Vav3*^–/–^ Astrocytes Enhance the Dendritic Development of Wildtype Neurons *in vitro*

**FIGURE 1:**
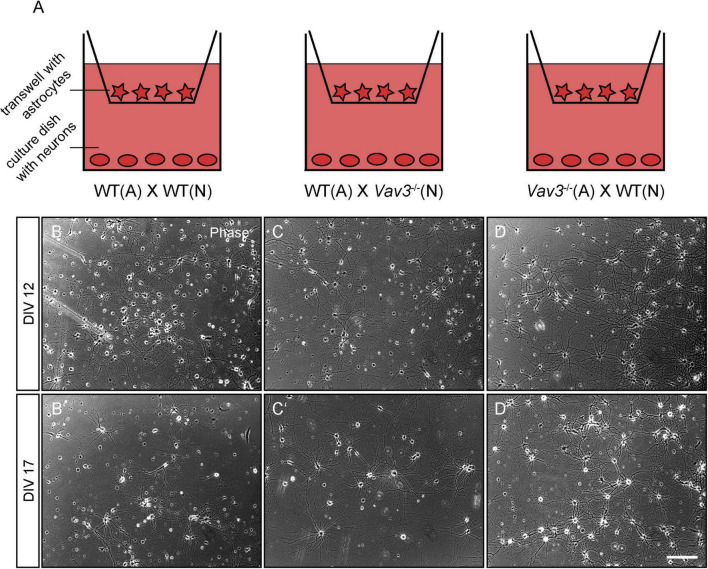
**(A)** Primary hippocampal neurons were cultured in an indirect co-culture setup with cortical astrocytes. In order to analyze the influence of a Vav3 deficiency in neurons and astrocytes, different combinations of *Vav3* knockout neurons and astrocytes were used. First, wildtype neurons were cultured with wildtype astrocytes and served as the control condition. Furthermore, *Vav3*^–/–^ neurons were combined with wildtype astrocytes for the analysis of neuron-specific effects induced by the *Vav3* knockout. Last, wildtype neurons were cultured with *Vav3*^–/–^ astrocytes to investigate possible positive or negative effects for native neurons. **(B–D)** Neurons were kept in culture for 12 days in completely defined medium without serum and formed a highly complex network. Native neurons cultured with *Vav3*^–/–^ astrocytes appeared slightly more complex in comparison to the other culture combinations. **(B’–D’)** Additionally, neurons were cultured for a longer culturing time of 17 days since the dendrites become longer and more complex at this point in time. Scale bar: 200 μm.

In order to analyze the impact of *Vav3*^–/–^ astrocytes on the development of primary hippocampal neurons, indirect co-cultures were used with varying combinations of wildtype/*Vav3*^–/–^ neurons and wildtype/*Vav3*^–/–^ astrocytes (Figure 1A). As the control condition, wildtype neurons were co-cultured with wildtype astrocytes. Furthermore, wildtype neurons were cultured in an indirect contact with *Vav3*^–/–^ astrocytes. Finally, *Vav3*^–/–^ neurons were kept in culture with wildtype astrocytes since it is known that a deficiency of *Vav3*^–/–^ influences the early development of axons and dendrites *in vitro* (Wegrzyn et al., 2020). The neurons of all three culture combinations formed highly branched and complex networks after a cultivation time of 12 and 17 days *in vitro* (Figures 1B–D,1B’–D’). The phase contrast images did not show obvious differences between the culture combinations at both analyzed timepoints.

**FIGURE 2:**
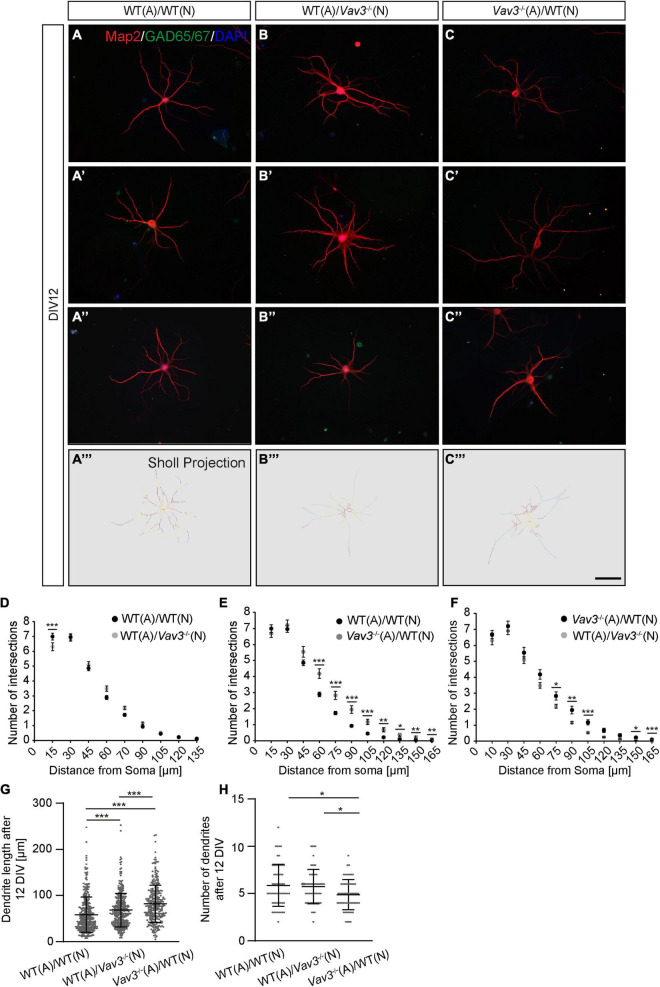
**(A–A”, B–B”, C–C”)** After a culturing time of 12 days neurons were immunocytochemically stained with specific antibodies against the dendritic marker Map2 and the interneuron marker GAD65/67. Furthermore DAPI was used for the visualization of nuclei. The GAD65/67 positive neurons were excluded from the analysis since interneurons have a highly differing morphology in comparison to pyramidal neurons and might falsify the experimental outcome. Three exemplary neurons are depicted per condition. **(A”’–C”’)** A Sholl analysis was performed for determining the dendritic complexity. The projections depict the skeletonized dendrites and red areas represent a high number of intersections while blue regions represent a low number of intersections, **(D–F)** The quantification of the Sholl analysis could show that especially wildtype neurons cultured with *Vav3*^–/–^ astrocytes developed more complex dendrites than neurons of both other conditions. **(G)** The quantification of the dendritic lengths revealed a significant increase in *Vav3*^–/–^ neurons cultured with wildtype astrocytes and in native neurons cultured with *Vav3*^–/–^ astrocytes in comparison to the controls. Dots are representing single analyzed dendrites. **(H)** In contrast, the number of dendrites was slightly reduced in the cultures consisting of wildtype neurons combined with *Vav3*^–/–^ astrocytes. Dots are representing individual neurons analyzed regarding their number of dendrites. Statistics: For the Sholl analysis five experimental repetitions (*N* = 5) were performed for the culture combinations WT(N)/WT(A) and *Vav3*^–/–^ (N)/WT(A) while three experimental repetitions (*N* = 3) were performed for the combination WT(N)/*Vav3*^–/–^ (A). Furthermore, 40 neurons (*n* = 40) were recorded and analyzed per experimental repetition. For the dendritic lengths and numbers 3 experimental repetitions (*N* = 3) were performed with 20 quantified neurons (*n* = 20) per repetition. Kruskal-Wallis test with a following Dunn’s multiple comparison test; *p ≤ 0.05, **p ≤ 0.01 and ***p ≤ 0.001, data are represented as mean ± SEM. Scale bar: 50 μm.

The analysis of the dendritic complexity was performed by an immunocytochemical staining against the microtubule associated protein Map2 which was subsequently combined with the Sholl analysis, as similarly performed in various studies (Figures 2A–A”,B–B”,C–C”, 3A–A”,B–B”,C–C”; Huang et al., 2011; Bartelt-Kirbach et al., 2016; Jacobs et al., 2016; Czaniecki et al., 2019). The expression of Map2 is strictly limited to the dendritic compartment and occurs in the main stable dendrites (Caceres et al., 1984). In addition, neurons were co-stained with an antibody against GAD65/67 because interneurons highly differ in their morphology and might negatively affect the outcome of the analysis. In this way, a differentiation between pyramidal neurons and interneurons was possible. The complexity of the dendritic trees was quantified using the Sholl analysis (Figures 2A”’–C”’, 3A”’–C”’; Sholl, 1953). After a cultivation time of 12 days *in vitro*, *Vav3*^–/–^ neurons cultured with wildtype astrocytes showed a mild but significantly decreased number of intersections in a region close to the cell soma compared to the control condition (Figure 2D). The number of intersections measured at all other distances from the soma revealed no significant changes between these two conditions. Table 2 gives an overview about all values.

**FIGURE 3:**
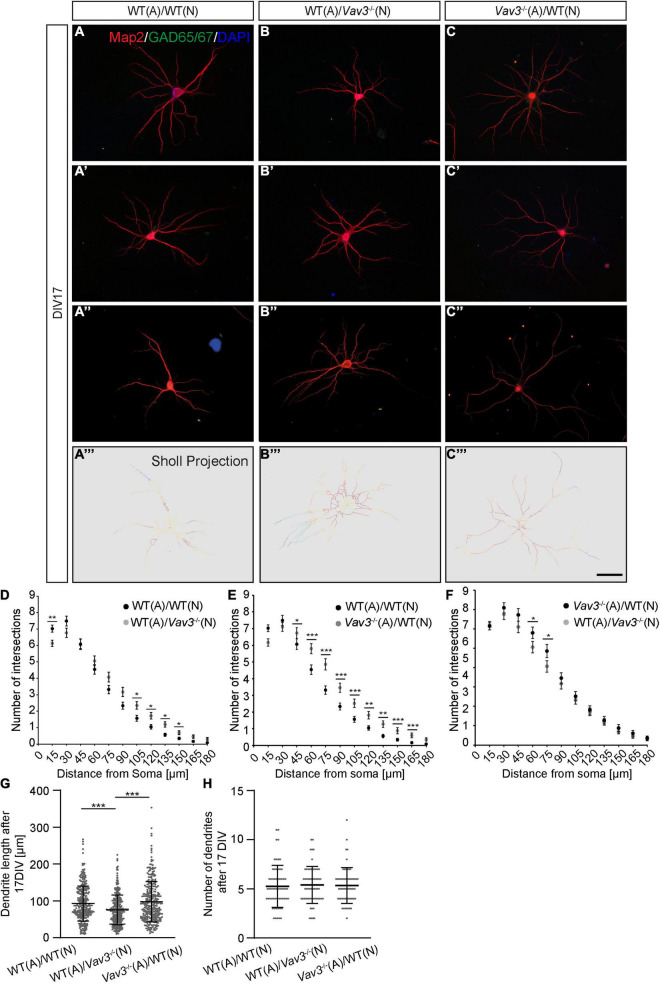
**(A–A”,B–B”,C–C”)** Neurons of the different combinations were fixed after a culturing time of 17 days and immunostained against Map2 and GAD65/67. As previously mentioned GAD65/67 positive neurons were excluded from the analysis since interneurons possess a strongly differing morphology compared to pyramidal neurons. DAPI was utilized for the visualization of nuclei. Three exemplary neurons are shown per condition. **(A”’–C”’)** The Sholl projection represents the skeletonized dendrites and depicts highly branched regions with a red labeling while less branched regions are marked by a blue labeling. **(D–F)** After a culturing time of 17 days *in vitro* wildtype neurons which were kept in culture with *Vav3*^–/–^ astrocytes showed a significantly increase in the number of intersections at nearly all analyzed distances from the soma. Furthermore, *Vav3*^–/–^ neurons developed a higher number of intersections in the distal part of the dendrites. **(G)** *Vav3*^–/–^ neurons cultured with wildtype astrocytes showed a significantly reduced dendritic length in comparison to both other conditions. Dots are representing single analyzed dendrites. **(H)** The quantification of the number of dendrites revealed no significant differences between all analyzed conditions. Dots are representing individual neurons analyzed for their number of dendrites. As already described in Figure 2 five experimental repetitions (*N* = 5) were performed for the culture combinations WT(N)/WT(A) and *Vav3*^–/–^(N)/WT(A) while three experimental repetitions (*N* = 3) were performed for the combination WT(N)/*Vav3*^–/–^(A) for the Sholl analysis. Furthermore, 40 neurons (*n* = 40) were recorded and analyzed per experimental repetition. For the dendritic lengths and numbers 3 experimental repetitions (*N* = 3) were performed with 20 quantified neurons (*n* = 20) per repetition. Kruskal-Wallis test with a following Dunn’s multiple comparison test; *p ≤ 0.05, **p ≤ 0.01 and ***p ≤ 0.001, data are represented as mean ± SEM. Scale bar: 50 μm.

**TABLE 2:** Intersection numbers of *Vav3*^–/–^ neurons compared to the control condition after a cultivation time of 12 days.

Distance from soma (μm)	Number of intersections WT(N) × WT(A)	Number of intersections *Vav3*^–/–^(N) × WT(A)	*p*-value
15	6.99 ± 0.17	6.31 ± 0.22	0.0004
30	6.96 ± 0.23	6.92 ± 0.26	0.982
45	4.86 ± 0.21	5.09 ± 0.24	>0.999
60	2.89 ± 0.17	3.49 ± 0.22	0.253
75	1.72 ± 0.13	2.19 ± 0.17	0.312
90	0.93 ± 0.11	1.17 ± 0.12	0.253
105	0.46 ± 0.08	0.53 ± 0.07	0.813
120	0.22 ± 0.05	0.26 ± 0.04	0.492
135	0.11 ± 0.04	0.14 ± 0.03	0.739

The most prominent difference could be seen when wildtype neurons cultured with *Vav3*^–/–^ astrocytes were compared with the control condition (Figure 2E). Here, *Vav3*^–/–^ astrocytes significantly increased the dendritic complexity of wildtype neurons between 60 and 165 μm distant from the soma compared to the control combination of wildtype neurons with wildtype astrocytes, as indicated in Table 3.

**TABLE 3:** Intersection numbers of wildtype neurons cultured with *Vav3*^–/–^ astrocytes in comparison to the control condition after cultivation time of 12 days.

Distance from soma (μm)	Number of intersections WT(N) × WT(A)	Number of intersections WT (N) × *Vav3*^–/–^(A)	*p*-value
15	6.99 ± 0.17	6.69 ± 0.26	0.534
30	6.96 ± 0.23	7.21 ± 0.31	>0.999
45	4.86 ± 0.21	5.55 ± 0.33	0.415
60	2.89 ± 0.17	4.18 ± 0.29	0.0005
75	1.72 ± 0.13	2.83 ± 0.25	0.0003
90	0.93 ± 0.11	1.95 ± 0.24	≤0.0001
105	0.46 ± 0.08	1.17 ± 0.16	≤0.0001
120	0.22 ± 0.05	0.68 ± 0.13	<0.0001
135	0.11 ± 0.04	0.37 ± 0.10	0.004
150	0.04 ± 0.02	0.27 ± 0.07	0.002
165	0.02 ± 0.01	0.10 ± 0.03	0.029

A comparison of wildtype neurons cultured with *Vav3*^–/–^ astrocytes with *Vav3*^–/–^ neurons cultured with wildtype astrocytes revealed only minor differences in a distance of 75, 90, and 105 μm (Figure 2F). The number of intersections and the *p*-values are listed in Table 4.

**TABLE 4:** Intersection numbers of wildtype neurons cultured with *Vav3*^–/–^ astrocytes in comparison to *Vav3*^–/–^ neurons cultured with wildtype astrocytes after cultivation time of 12 days.

Distance from soma (μm)	Number of intersections *Vav3*^–/–^ (N) × WT(A)	Number of intersections WT (N) × *Vav3*^–/–^ (A)	*p*-value
15	6.31 ± 0.22	6.69 ± 0.26	0.147
30	6.92 ± 0.26	7.21 ± 0.31	0.566
45	5.09 ± 0.24	5.55 ± 0.33	0.558
60	3.49 ± 0.22	4.18 ± 0.29	0.068
75	2.19 ± 0.17	2.83 ± 0.25	0.042
90	1.17 ± 0.12	1.95 ± 0.24	0.008
105	0.53 ± 0.07	1.17 ± 0.16	0.0007
120	0.26 ± 0.04	0.68 ± 0.13	0.173
135	0.14 ± 0.03	0.37 ± 0.10	0.182
150	0.06 ± 0.02	0.27 ± 0.07	0.019
165	0.02 ± 0.02	0.10 ± 0.03	0.0005

In addition to the Sholl analysis, the average number of dendrites per neuron and the dendritic lengths were analyzed (Figures 2G,H). Wildtype neurons which were co-cultured with wildtype astrocytes formed dendrites with an average length of 57.79 ± 2.03 μm after a cultivation time of 12 days. Interestingly, *Vav3*^–/–^ neurons cultured with wildtype astrocytes formed significantly longer dendrites with an average length of 68.26 ± 4.71 μm when they were compared with the previously mentioned control condition (*p* < 0.0001). However, the longest dendrites could be observed when wildtype neurons were co-cultured with *Vav3*^–/–^ astrocytes. In this combination, neurons formed dendrites with an average length of 82.02 ± 5.22 μm and were significantly longer in comparison to both other conditions (*p* < 0.0001) (Figure 2G). For the number of dendrites a slight but significant (*p* = 0.014) reduction could be seen when wildtype neurons were cultured with *Vav3*^–/–^ astrocytes (4.88 ± 0.21) and compared with the control condition (5.82 ± 0.29) (Figure 2H).

The dendritic parameters were furthermore analyzed after a longer cultivation time of 17 days *in vitro* (Figures 3A–A”,B–B”,C–C”). Here, *Vav3*^–/–^ neurons cultured with wildtype astrocytes showed a mild increase in the number of intersections in 105–150 μm distant from the soma compared to the control condition (Figure 3). Table 5 contains all values of the afore mentioned combinations.

**TABLE 5:** Intersection numbers of *Vav3*^–/–^ neurons in comparison to the control condition after cultivation time of 17 days.

Distance from soma (μm)	Number of intersections WT(N) × WT(A)	Number of intersections *Vav3*^–/–^ (N) × WT(A)	*p*-value
15	7.03 ± 0.21	6.13 ± 0.18	0.0049
30	7.49 ± 0.30	6.76 ± 0.28	0.240
45	6.07 ± 0.32	6.10 ± 0.29	> 0.999
60	4.55 ± 0.29	5.06 ± 0.29	0.490
75	3.32 ± 0.25	4.07 ± 0.30	0.161
90	2.34 ± 0.22	3.17 ± 0.27	0.057
105	1.57 ± 0.18	2.35 ± 0.23	0.047
120	1.05 ± 0.14	1.72 ± 0.20	0.022
135	0.57 ± 0.09	1.19 ± 0.16	0.043
150	0.34 ± 0.08	0.69 ± 0.12	0.021
165	0.17 ± 0.05	0.46 ± 0.10	0.061
180	0.09 ± 0.03	0.31 ± 0.09	0.099

In accordance with the previously described increase of the dendritic complexity when wildtype neurons were cultured with *Vav3*^–/–^ astrocytes for 12 days *in vitro*, similar results were obtained after a longer cultivation time (Figure 3E). Here, the neurons formed dendrites with a clearly increased number of intersections at nearly all analyzed distances. The exact values are given in Table 6.

**TABLE 6:** Intersection numbers of wildtype neurons cultured with *Vav3*^–/–^ astrocytes in comparison to the control condition after cultivation time of 17 days.

Distance from soma (μm)	Number of intersections WT(N) × WT(A)	Number of intersections WT (N) × *Vav3*^–/–^ (A)	*p*-value
15	7.03 ± 0.21	6.17 ± 0.22	0.092
30	7.49 ± 0.30	7.10 ± 0.26	> 0.999
45	6.07 ± 0.32	6.73 ± 0.33	0.026
60	4.55 ± 0.29	5.80 ± 0.30	0.0002
75	3.32 ± 0.25	4.86 ± 0.39	< 0.0001
90	2.34 ± 0.22	3.46 ± 0.27	0.0001
105	1.57 ± 0.18	2.52 ± 0.25	0.0003
120	1.05 ± 0.14	1.81 ± 0.22	0.002
135	0.57 ± 0.09	1.28 ± 0.19	0.003
150	0.34 ± 0.08	0.88 ± 0.18	0.0004
165	0.17 ± 0.05	0.61 ± 0.14	0.0008
180	0.09 ± 0.03	0.36 ± 0.10	0.055

When wildtype neurons cultured with *Vav3*^–/–^ astrocytes were compared with *Vav3*^–/–^ neurons cultured with wildtype astrocytes only minor differences were seen after 17 DIV (Figure 3F). In a distance of 60 and 75 μm from the soma *Vav3*^–/–^ neurons formed a higher number of intersections. However, all other analyzed distances revealed no significant alteration of the intersection number. The whole data set is listed in Table 7.

**TABLE 7:** Intersection numbers of wildtype neurons cultured with *Vav3*^–/–^ astrocytes in comparison to *Vav3*^–/–^ neurons cultured with wildtype astrocytes after cultivation time of 17 days.

Distance from soma (μm)	Number of intersections *Vav3*^–/–^ (N) × WT(A)	Number of intersections WT (N) × *Vav3*^–/–^ (A)	*p*-value
15	6.13 ± 0.18	6.17 ± 0.22	> 0.999
30	6.76 ± 0.28	7.10 ± 0.26	0.174
45	6.10 ± 0.29	6.73 ± 0.33	0.070
60	5.06 ± 0.29	5.80 ± 0.30	0.016
75	4.07 ± 0.30	4.86 ± 0.39	0.022
90	3.17 ± 0.27	3.46 ± 0.27	0.103
105	2.35 ± 0.23	2.52 ± 0.25	0.218
120	1.72 ± 0.20	1.81 ± 0.22	0.850
135	1.19 ± 0.16	1.28 ± 0.19	0.685
150	0.69 ± 0.12	0.88 ± 0.18	0.400
165	0.46 ± 0.10	0.61 ± 0.14	0.312
180	0.31 ± 0.09	0.36 ± 0.10	> 0.999

The dendritic lengths as well as the number of dendrites were also analyzed after 17 DIV (Figures 3G,H). Here, the increased dendrite lengths of wildtype neurons cultured with *Vav3*^–/–^ astrocytes could not be observed anymore. Neurons of the control condition formed dendrites with an average length of 92.97 ± 2.65 μm. In comparison, wildtype neurons cultured with *Vav3*^–/–^ astrocytes formed dendrites with a similar average dendritic length of 97.70 ± 7.07 μm. The statistical test revealed no significance for these two conditions (*p* > 0.999). Interestingly, *Vav3*^–/–^ neurons co-cultured with wildtype astrocytes developed dendrites with an average length of 75.41 ± 5.21 μm and were significantly shorter compared to both other conditions (*p* < 0.0001) (Figure 3G). With regard to the number of dendrites no significant change could be seen when all three combinations were compared with each other (Figure 3H).

### *Vav3^–/–^* Astrocytes Show Higher Expression Levels of *NT-3* and *TSP-1 in vitro*

**FIGURE 4:**
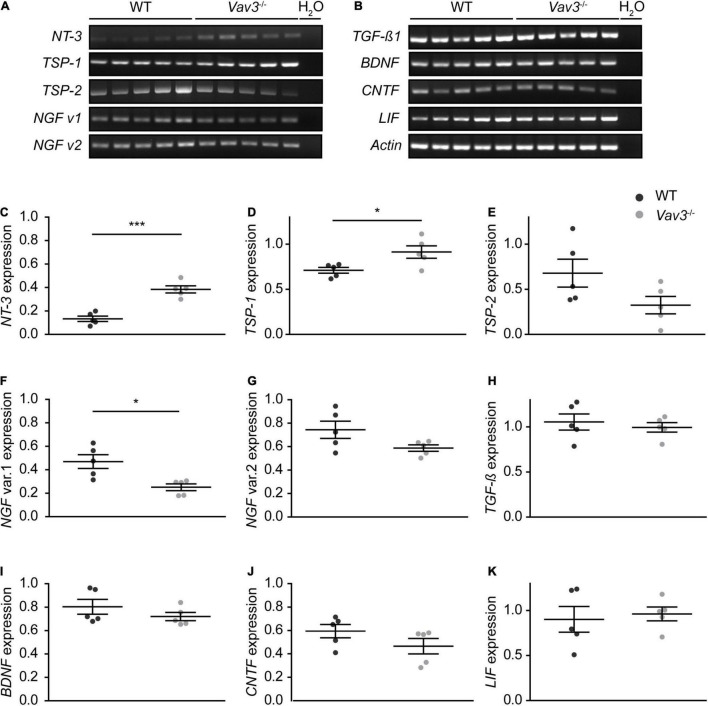
**(A,B)** Wildtype and *Vav3*^–/–^ astrocytes were lysed and a RT-PCR analysis with primers for different neurotrophic factors was performed. The signal intensity of *NT-3* appeared stronger in *Vav3*^–/–^ astrocytes compared to wildtype astrocytes. Furthermore, there was a slightly reduced signal intensity of the transcript variant 1 of *NGF* in *Vav3*^–/–^ astrocytes. **(C–K)** The signal intensity of the PCR bands was set in relation to the actin positive control and compared between both groups. The quantification revealed a significantly increased expression of *NT-3* and *TSP-1* while the transcript variant of *NGF* was reduced. The expression of all other analyzed neurotrophic factors was unchanged in the knockout condition. Individual dots are representing the signal intensity of every sample in relation to actin Statistics: in total, five independent experimental repetitions (*N* = 5) were performed and one lysed sample (*n* = 1) per repetition was used for the PCRs. Unpaired *t*-test; *p ≤ 0.05, **p ≤ 0.01 and ***p ≤ 0.001, data are represented as mean ± SEM.

Since a higher arborization and length of dendrites were observed when wildtype neurons were co-cultured with Vav3 deficient astrocytes, the expression levels of different neurotrophins and neurotrophic cytokines were investigated via RT-PCR analysis (Figures 4A,B). Interestingly, *Vav3*^–/–^ astrocytes showed a significantly raised expression of *NT-3* and *TSP-1* (Figures 4C,D). Here, wildtype neurons showed a relative expression of 0.13 ± 0.05 for *NT-3* and 0.71 ± 0.07 for *TSP-1*. In comparison, *Vav3*^–/–^ astrocytes displayed significantly increased expression levels for *NT-3* (0.38 ± 0.07, *p* = 0.0002) and *TSP-1* (0.91 ± 0.15, *p* = 0.0289). Surprisingly, NT-3 could merely be detected in exceedingly low levels in the conditioned medium of wildtype and *Vav3*^–/–^ astrocytes via ELISA (Supplementary Figure 1). A comparison of the OD_450_ values revealed similar signals intensities between both analyzed conditions. Furthermore, the expression of *thrombospondin-2* (*TSP-2*) was unchanged when both groups were compared with each other (WT: 0.68 ± 0.34, *Vav3*^–/–^: 0.32 ± 0.21, *p* = 0.868) (Figure 4E). A significant downregulation of the transcript variant 1 of the *nerve growth factor* (*NGF*) could be seen in *Vav3*^–/–^ astrocytes (0.25 ± 0.06) compared to wildtype astrocytes (0.47 ± 0.13, *p* = 0.01) (Figure 4F). A comparison of the expression levels of further neurotrophic factors revealed no significant alterations (Figures 4G–K). Here, the expression of the transcript variant 2 of *NGF* (WT: 0.74 ± 0.16, *Vav3*^–/–^: 0.59 ± 0.06, *p* = 0.082), *transforming growth factor*β (*TGF-*β) (WT: 1.05 ± 0.19, *Vav3*^–/–^: 0.99 ± 0.12, *p* = 0.578), *brain-derived neurotrophic factor* (*BDNF*) (WT: 0.80 ± 0.14, *Vav3*^–/–^: 0.72 ± 0.08, *p* = 0.283), *ciliary neurotrophic factor* (*CNTF*) (WT: 0.59 ± 0.13, *Vav3*^–/–^: 0.47 ± 0.15, *p* = 0.176) and *leukemia inhibitory factor* (*LIF*) (WT: 0.90 ± 0.32, *Vav3*^–/–^: 0.96 ± 0.17, *p* = 0.716) was similar in wildtype and *Vav3*^–/–^ astrocytes.

### *Vav3*^–/–^ Astrocytes Show an Altered Secretion Pattern of Chemokines and Cytokines

**FIGURE 5:**
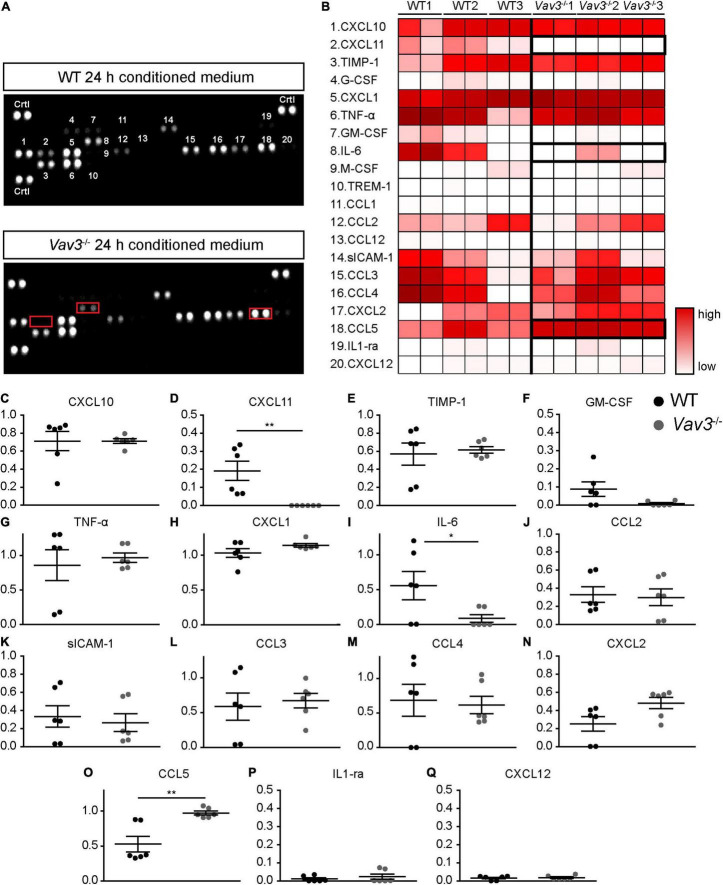
**(A)** Serum-free neuronal medium was conditioned for 24 h with the secreted factors of wildtype and *Vav3*^–/–^ astrocytes. Then, cytokine arrays were used in order to determine the cytokine and chemokine profiles of both groups. The conditioned medium of *Vav3*^–/–^ astrocytes revealed an altered profile with a lack of CXCL11, reduced levels of IL-6 and increased levels of CCL5 (indicated by red boxes). **(B)** A total number of 20 cytokines and chemokines could be detected in the supernatants and are depicted in the heatmap. The individual numbers on the membranes are defined by the corresponding cytokine and chemokine names in the heatmap. A low cytokine level is represented by a white color while a strong level is represented by dark red colors. As indicated by the bold boxes, the conditioned medium of *Vav3*^–/–^ astrocytes showed a lack of CXCL11, increased concentrations of CCL5 and reduced levels of IL-6. **(C–Q)** The statistical analysis of the cytokine array confirmed these observations. Since the concentrations of Trem1, CCL1, G-CSF, M-CSF, and CCL12 were exceedingly low in the supernatants they were shown in detail by diagrams. The exact values can be found in the result part 3.3. Statistics: Three experimental repetitions (*N* = 3) were performed, and two technical replicates (*n* = 2) were considered per repetition. Unpaired *t*-test, *p ≤ 0.05, **p ≤ 0.01 and ***p ≤ 0.001, data are represented as mean ± SEM.

Because NT-3 could not be detected in the conditioned medium of wildtype as well as *Vav3*^–/–^ astrocytes, a cytokine array analysis was performed in order to identify further secreted factors which might possibly be responsible for the enhanced dendrite growth and branching (Figure 5A). For this purpose, serum free medium with the same composition as the neuronal culture medium described under 2.2.5 was conditioned for 24 h by wildtype and *Vav3*^–/–^ astrocytes. The cytokine array revealed similar values for most of the cytokines detected in the supernatants, as indicated in the heatmap (Figure 5B). However, the analysis of the conditioned medium of *Vav3*^–/–^ astrocytes unraveled reduced levels of IL-6 and a complete lack of CXCL11 (Figures 5A,B). Furthermore, the conditioned medium of *Vav3*^–/–^ astrocytes showed an increased concentration of CCL5. The statistical quantification of the data verified these observations (Figures 5C–Q). For CXCL11, a relative signal intensity of 0.19 ± 0.05 could be detected in the supernatant of wildtype astrocytes while the signal was nearly not detectable in the supernatant of *Vav3*^–/–^ astrocytes [0.00004 ± 0.00002 (*p* = 0.0049)] (Figures 5B,D). Further members of the CXCL chemokine family could be observed but did not show significant alterations between both conditions (CXCL1: WT: 1.03 ± 0.064, *Vav3*^–/–:^ 1.14 ± 0.025, *p* = 0.136; CXCL2: WT: 0.25 ± 0.080, *Vav3*^–/–:^ 0.48 ± 0.06, *p* = 0.093; CXCL10: WT: 0.71 ± 0.10, *Vav3*^–/–^: 0.71 ± 0.03, *p* = 0.999; CXCL12: WT: 0.015 ± 0.004, *Vav3*^–/–:^ 0.018 ± 0.004, *p* = 0.818) (Figures 5B,C,H,N,Q). Interestingly, the conditioned medium of *Vav3*^–/–^ astrocytes showed a reduced signal intensity for IL-6 which was 0.09 ± 0.05 and hence significantly reduced in comparison to the signal intensity of IL-6 in the supernatant of wildtype astrocytes [0.56 ± 0.20 (*p* = 0.0488)] (Figures 5B,I). Beside IL-6, the cytokine TNF-α (WT: 0.86 ± 0.22, *Vav3*^–/–:^ 0.96 ± 0.07, *p* = 0.650) and IL-1ra (WT: 0.01 ± 0.006, *Vav3*^–/–:^ 0.02 ± 0.014, *p* = 0.999) could be detected without significant changes between wildtype and *Vav3*^–/–^ astrocytes (Figures 5B,G,P). CCL5 was the only detected cytokine which showed a significantly increased signal intensity in the knockout condition. Here, an average signal intensity of 0.53 ± 0.11 could be seen in the supernatant of wildtype astrocytes and 0.97 ± 0.029 in the supernatant of *Vav3*^–/–^ astrocytes (*p* = 0.003) (Figures 5B,O). In addition, several other members of the CCL chemokine family were verified in similar levels between both conditions (CCL1: WT: 0.004 ± 0.002, *Vav3*^–/–:^ 0.003 ± 0.002, *p* = 0.686; CCL2: WT: 0.33 ± 0.09, *Vav3*^–/–:^ 0.30 ± 0.09, *p* = 0.809; CCL3: WT: 0.59 ± 0.20, *Vav3*^–/–^: 0.67 ± 0.11, *p* = 0.706; CCL4: WT: 0.68 ± 0.23, *Vav3*^–/–:^ 0.61 ± 0.13, *p* = 0.802; CCL12: 0.006 ± 0.003, *Vav3*^–/–:^ 0.004 ± 0.002, *p* = 0.700) (Figures 5B,J,L,M). Different colony stimulating factors could be verified in the supernatant of both, wildtype and knockout astrocytes. However, their signal intensities were low, and no significant changes could be seen (G-CSF: WT: 0.04 ± 0.016, *Vav3*^–/–:^ 0.01 ± 0.005, *p* = 0.115; GM-CSF: WT: 0.088 ± 0.04, *Vav3*^–/–:^ 0.009 ± 0.005, *p* = 0.080; M-CSF: WT: 0.032 ± 0.017, *Vav3*^–/–:^ 0.020 ± 0.010, *p* = 0.559) (Figures 5B,F). Finally, Timp-1 (WT: 0.57 ± 0.122, *Vav3*^–/–:^ 0.61 ± 0.04, *p* = 0.724), sICAM-1 (WT: 0.33 ± 0.120, *Vav3*^–/–:^ 0.26 ± 0.097, *p* = 0.670) and TREM-1 (WT: 0.003 ± 0.002, *Vav3*^–/–:^ 0.03 ± 0.001, *p* = 0.802) were detected with similar mean values between both analyzed groups (Figures 5B,E,K).

### *Vav3*^–/–^ Astrocytes Show a Faster Regeneration in a Scratch Wound Healing Assay

**FIGURE 6:**
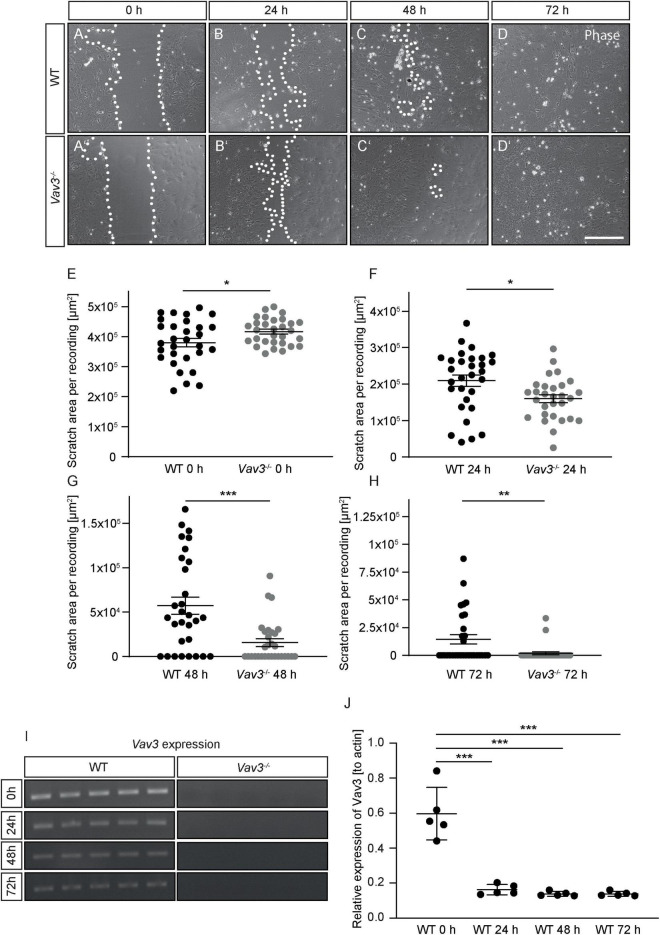
**(A–D,A’–D’)** Wildtype and *Vav3*^–/–^ astrocytes were tested regarding their migration behavior in a scratch wound healing assay. Phase contrast images were taken immediately after performing the scratch (0 h) and after 24, 48, and 72 h. **(E–H)** The scratch area was visibly smaller in the *Vav3*^–/–^ astrocyte cultures after 24 and 72 h, although the initially performed scratch was greater in these cultures. **(I)** RT-PCR analysis confirmed the absence of *Vav3*^–/–^ in cultured astrocytes. **(J)** Furthermore, the expression of *Vav3* was significantly reduced in wildtype cultures after performing the scratch. Statistics: In total, five experimental repetitions (*N* = 5) were performed for the scratch wound healing assay. Here, 6 phase contrast images (*n* = 6) were recorded and quantified per experimental repetition. For the *Vav3* RT-PCR also five experimental repetitions (*N* = 5) and one sample per preparation (*n* = 1) were used. Unpaired *t*-test, *p ≤ 0.05, **p ≤ 0.01 and ***p ≤ 0.001, data are represented as mean ± SEM. Scale bar: 200 μm.

Rho-GTPases are highly involved in the regulation of cytoskeletal reorganizations. This is necessary in cases of migration and proliferation. In order to examine if a lack of Vav3 has effects on the astrocytic migration, a scratch wound healing assay was performed (Etienne-Manneville, 2006; Figures 6A–D,A’–D’). Although the average size of the initial scratch was higher in the *Vav3*^–/–^ astrocyte cultures (416987.73 ± 7963.12 μm^2^) than in the wildtype cultures (380123.67 ± 14206.58 μm^2^, *p* = 0.028), *Vav3*^–/–^ astrocytes showed a significantly lower scratch area after 24 h (WT: 208997.23 ± 15449.84 μm^2^, *Vav3*^–/–^: 159452.72 ± 10843.95 μm^2^, *p* = 0.016), 48 h (WT: 57346.53 ± 9698.27 μm^2^, *Vav3*^–/–^: 15674.60 ± 4302.31 μm^2^, *p* = 0.0002) and 72 h (WT: 14496.64 ± 4273.20 μm^2^, *Vav3*^–/–^: 1872.90 ± 1325.65 μm^2^, *p* = 0.0065) (Figures 6F–H). A lack of Vav3 in the knockout cultures was verified via RT-PCR analysis (Figure 6I). Interestingly, a scratch in the wildtype astrocyte monolayer induced the downregulation of Vav3 on mRNA level as shown in Figure 6J. Before performing the scratch, native astrocytes showed a relative expression of 0.59 ± 0.15 for *Vav3*. However, 24 h after the scratch the astrocytes showed just a relative *Vav3* expression of 0.16 ± 0.03. Similar results were also obtained 48 h (0.14 ± 0.01) and 72 h (0.14 ± 0.01) after the scratch. The relative expression of Vav3 at all analyzed points in time after the scratch was significantly reduced (*p* < 0.0001).

## Discussion

### Vav3 Deficiency Alters Dendrite Lengths of Hippocampal Neurons After 12 and 17 DIV

In the present study, we could observe that a knockout of the GEF Vav3 has merely mild effects on the dendritic complexity of primary hippocampal neurons *in vitro* (Figures 2D, 3D). After a culturing time of 12 days nearly no difference was visible between wildtype and knockout neurons, while after 17 days a mild increase in the number of intersections could be detected. This was especially the case for the distal regions of *Vav3*^–/–^ dendrites. Nevertheless, the lack of Vav3 showed a stronger impact on the average dendritic length. Here, a statistically significant increase of the dendrite lengths was observed after 12 days *in vitro* and a decrease after 17 days *in vitro* (Figures 2G, 3G). These data suggest, that Vav3 rather acts as a regulator of the dendrite elongation than as a regulator of the dendritic branching, at least, in the here analyzed culture period of 17 days. In a previous study, we could already observe an increase of the dendrite lengths after a short culturing time of 3 and 5 days in primary hippocampal neurons lacking Vav3 (Wegrzyn et al., 2020). This indicates that Vav3 might have distinct regulatory roles for the early and later dendrite development. Interestingly, a previous study could prove an important function of Vav3 for the dendrite branching of Purkinje and granule cells in the cerebellum of mice (Quevedo et al., 2010). Here, significant branching deficits could be seen in the early postnatal period which vanished in adult animals. Furthermore, this study could detect varying expression levels of Vav3 in different developmental stages, supporting the previously mentioned aspect of an regulating function of Vav3 during early and late developmental stages or culturing periods (Quevedo et al., 2010). Other studies unraveled significant effects of a *Vav3* knockout on the axonal development in early culturing stages as well as *in vivo* (Sauzeau et al., 2010; Wegrzyn et al., 2020). Because of the highly complex wiring of individual axons with axons of other neurons, this neuronal compartment was not considered for this study. Nevertheless, it would be of great interest to investigate the impact of a Vav3 deletion on the axonal morphology in matured neurons in future studies. Here, cultures with a low cell density could be used to track individual axons. As indicated in the introduction, Vav3 acts as an activator of the Rho-GTPases RhoA, Rac-1 and Cdc42, which are modulators of the cytoskeleton (Movilla and Bustelo, 1999; Movilla et al., 2001; Scott et al., 2003; Aoki et al., 2005). Biochemical [^3^H]GDP-releasing assays could verify that Vav3 strongly induces a nucleotide exchange on RhoA, to a lesser extent on Rac-1 and is nearly inactive on Cdc42 (Movilla and Bustelo, 1999). Therefore, most of the observed effects in this study might result from a lowered RhoA activity. However, this assumption must be proven by analyzing the activity of the different Rho-GTPases in *Vav3* knockout neurons in future studies. Unfortunately, the low cell number of the present culture system did not permit for the detection of individual Rho-GTPase activities. Nevertheless, previous studies could already show that a lack of RhoA in neurons of *Drosophila melanogaster* was responsible for the overextension of dendrites (Lee et al., 2000). Here, the authors discuss that RhoA is essential for a limitation of the dendrite growth. In accordance with this conclusion, studies that utilized dominant active forms of RhoA revealed a reduction of dendrite lengths and a simplification of dendritic trees in rodents via Rho-associated protein kinase (ROCK) activation (Nakayama et al., 2000; Ahnert-Hilger et al., 2004). This is in accordance with our observation of the increased dendrite lengths after 12 days *in vitro* but not with the reduced dendrite lengths after 17 days *in vitro*. A possible explanation might be that compensatory mechanisms intervene after a longer culturing time and that other GEFs like Vav2 prevent a persisting extension of dendrites. Here, it has been shown that Vav2 unfolds regulatory functions for the branching and elongation of neurites in *Xenopus laevis in vivo* as well as *in vitro* (Moon and Gomez, 2010). Interestingly, a previous study described the protein expression levels of Vav2 in whole brain lysates (without cerebellar tissue) at different developmental stages and observed a strong expression at E13.5-E18.5 which gradually decreased at postnatal stages and reached the lowest levels in adult animals (Cowan et al., 2005). Since this study showed a gradual decrease over time it might be possible that Vav2 merely compensates the effects of a *Vav3* knockout on the dendritic complexity at early culturing stages but not in advanced cultures. Here, the slight increase of the intersection number in regions farther away from the soma at DIV17 as well as the decreased dendritic lengths might be indications for this. However, Vav2 does not prevent an overextension of dendrites at DIV12. For this reason it is necessary to define the expression levels of Vav2 also *in vitro*. Furthermore, there is a great number of GEFs which could be upregulated and compensate the lack of Vav3 explaining the mild phenotype. Here members of the ArhGEF family could play an important role. Recent studies unraveled that especially ArhGEF1 and ArhGEF2 are highly involved in the regulation of the dendritic development as activators of RhoA (Xiang et al., 2016, 2017; Zhou et al., 2021). Other potential candidates are Ephexins as well as Trio which have important regulatory functions for the dendritogenesis (Wu et al., 2007; Iyer et al., 2012). In addition, lowered activities of Rac-1 or Cdc42 could be further explanations for the observed effects. Studies utilizing dominant negative forms of Rac-1 or knockdowns revealed only mild changes for the dendrite complexity but a progressive elimination of dendritic spines in hippocampal neurons (Nakayama et al., 2000; Gualdoni et al., 2007). Therefore, a lowered Rac-1 activity as consequence of the *Vav3* knockout might not primarily affect the dendritic complexity but rather the spine number and stability. Furthermore, studies could show that a reduced or lacking Cdc42 activity induced the formation of more dendritic branches but impaired the stability of dendritic spines and synapses (Wegner et al., 2008; Schulz et al., 2016). Here, also a reduced Cdc42 activity induced by the lack of Vav3 might explain the slightly increased intersection number of Vav3-deficient neurons after 17 days *in vitro*. Since Rac1 and Cdc42 play an important role for the formation and stability of dendritic spines it would be of great interest to analyze the dendrite number and morphology in *Vav3*^–/–^ neurons.

### *Vav3*^–/–^ Astrocytes Promote the Dendritic Development of Wildtype Neurons and Show an Altered Cytokine Profile With Increased CCL5 Levels, Reduced IL-6 Levels and a Lack of CXCL11

The most prominent effect could be observed when wildtype neurons were co-cultured with *Vav3*^–/–^ astrocytes (Figures 2E,G, 3E,G). Here, wildtype neurons developed significantly longer dendrites and higher branched dendritic trees than neurons cultured with wildtype astrocytes. To determine how *Vav3*^–/–^ astrocytes enhance the elongation and branching of dendrites a RT-PCR analysis for different neurotrophic factors as well as a cytokine array with conditioned media of wildtype and knockout astrocytes was performed (Figures 4, 5). It is well known that astrocytes secrete a variety of neurotrophic factors and promote therefore the neurite growth as well as the synaptogenesis (Müller et al., 1995; Clarke and Barres, 2013). NT-3 is one of these secreted factors and acts through the tropomyosin receptor kinase C (TrkC) promoting the outgrowth, branching and elongation of neurites (Rudge et al., 1992; Morfini et al., 1994; Labelle and Leclerc, 2000; Mele et al., 2010). The RT-PCR analysis revealed significantly raised expression levels of *NT-3* in *Vav3*^–/–^ astrocytes (Figure 4C). For further analysis we focused on the NT-3 protein levels in the conditioned supernatants of wildtype and *Vav3*^–/–^ astrocytes. Surprisingly, very low signal intensities could be observed for NT-3 when the ELISA was performed (Supplementary Figure 1). We suspect that the conditioning period of 24 h possibly was too short. A longer conditioning time might lead to a higher accumulation of NT-3 in the supernatant which should be better detectable. Nevertheless, if *Vav3*^–/–^ astrocytes would secrete significantly higher amounts of NT-3 differences between wildtype and knockout astrocytes should be seen, also after a short conditioning time. Therefore, it is questionable if NT-3 is responsible for the enhanced dendrite development observed in our experiments. TSP-1 is another astrocytic factor which is highly involved in synapse formation via integrin α3β1 signaling (O’Shea et al., 1991; Osterhout et al., 1992; DeFreitas et al., 1995; Christopherson et al., 2005). The RT-PCR analysis revealed significantly raised expression levels of *TSP-1* in *Vav3*^–/–^ astrocytes (Figure 4D). Since TSP-1 plays an important role for the synapse formation it would be interesting to analyze the influence of *Vav3*^–/–^ astrocytes on native neurons rather in the context of structural synapse numbers than in the context of dendrite development. Future studies might utilize the indirect co-culture system for this purpose and focus on the expression of synaptic proteins. Surprisingly, the expression of the transcript variant 1 of *NGF* was reduced in the RT-PCR analysis (Figure 4F). NGF normally acts as a strong promoter of the neurite outgrowth by activating the tropomyosin receptor kinase A (TrkC). Therefore, a reduced expression level of NGF should impair the dendrite elongation. Since this was not the case in our morphological analysis, we assume that increased levels of other secreted factors might dominate the lowered NGF levels and shift the dendrites toward extensions and branching mechanisms.

**FIGURE 7:**
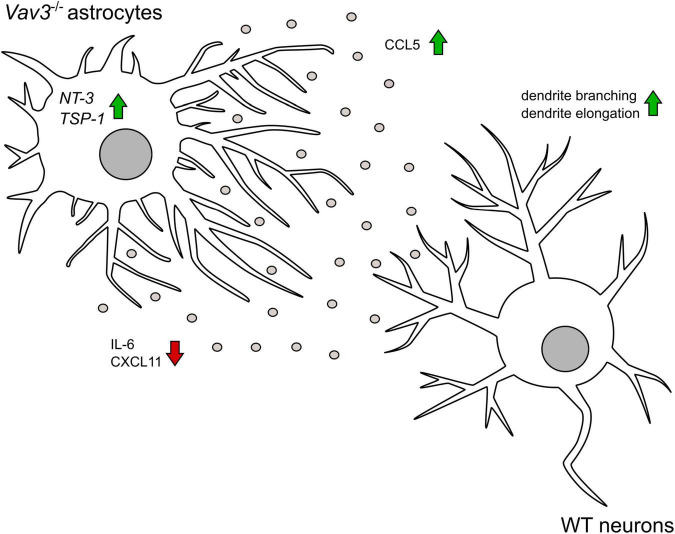
The graphical abstract depicts that *Vav3*^–/–^ astrocytes promote the branching and the elongation of dendrites on wildtype neurons in an indirect co-culture system. Knockout astrocytes show a higher secretion of the chemokine CCL5 which has a neurotropic potential. Contrary, the pro-inflammatory cytokine IL-6 is released in lower amounts. In contrast to wildtype astrocytes, *Vav3*^–/–^ astrocytes do not release CXCL11. On mRNA level *Vav3*^–/–^ astrocytes express more *NT-3* and *TSP-1*.

In addition to neurotrophic factors, astrocytes are able to express and secrete different cytokines and chemokines as previously described for humans and rodents (Wiese et al., 2012; Choi et al., 2014). A comparison between the cytokine patterns of wildtype and *Vav3*^–/–^ astrocytes revealed mainly three aberrances in the knockout condition: A complete lack of CXCL-11 which is also referred to as Interferon-inducible T-cell alpha chemoattractant (I-TAC), a significant reduction of IL-6 and a significant increase of CCL5 (Figures 5A,B,D,I,O). The most interesting candidate for the enhanced dendritic elongation and branching, is CCL5, also known as Regulated on Activation Normal T-cell Expressed and Secreted (RANTES). A recently published study could show that the treatment of hippocampal neurons with CCL5 activated the PI3K/Akt signaling pathway, which is an important pathway for the neurite outgrowth and elongation (Ajoy et al., 2021). Interestingly, this study could also show that an overexpression of CCL5 promotes the aerobic glucose metabolism, ATP synthesis, synapse formation, and enhances the memory in mice (Ajoy et al., 2021). Furthermore, in a Huntingtin model, the conditioned medium of primary astrocytes contained significantly lower levels of CCL5 and the treatment of cortical neurons induced a reduction of the neurite length and branching (Chou et al., 2008). Therefore, CCL5 is discussed to be a promising neurotrophic factor and could already show beneficial effects in a recently published optic nerve regeneration study (Xie et al., 2021). As previously mentioned, *Vav3*^–/–^ astrocytes showed a lack of CXCL11. Since there are no data about the influence of CXCL11 on the dendritic development of neurons, it is difficult to speculate about the consequences of a CXCL11 lack in our co-cultures. A previously published study could observe that CXCL11 induces the death of dopaminergic neurons in midbrain neuron-glia mixed cultures but not in enriched neuronal cultures, indicating an indirect toxic effect on neurons (Chien et al., 2016). Furthermore, it is known that CXCL11 acts through the binding to CXCR3, a Gα-protein-coupled receptor. An activation of CXCR3 with CXCL10 which acts similarly as CXCL11 was associated with an influx of Ca^2+^, the activation of caspases and apoptosis (Sui et al., 2006). Contrary, an inhibition of CXCR3 unraveled positive effects for the neuronal survival in different disease models (Zhang et al., 2010; van Weering et al., 2011). A lack of CXCL11 might therefore induce neuroprotective effects and enhance the development of neurites, however, further studies are necessary to shed light on the influence of CXCL11 on dendritic parameters. Last, the IL-6 levels were significantly lower in the conditioned medium of *Vav3*^–/–^ astrocytes. Here, a study on cultured hippocampal neurons revealed that a treatment with increasing levels of IL-6 increased the length of secondary neurites (Sarder et al., 1996). However, the number of branch points of the primary dendrite per neuron remained unaltered and a detailed number about clearly defined dendritic branches is not given. Another study revealed that high levels IL-6 inhibit the dendrite growth and branching in primary cortical cultures (Gilmore et al., 2004). A reduced secretion of IL-6 might therefore rather enhance the dendritic development. The graphical abstract (Figure 7) summarizes a suggestion how the knockout of *Vav3* in astrocytes might affect dendritic parameters of neurons.

### A Lack of Vav3 Promotes the Regeneration of Astrocytes in the Scratch Wound Healing Assay

One remaining question is how a deficiency of Vav3 induces an altered profile of released chemokines and cytokines. A possible explanation might be that a reduced activity of RhoA which is the main target of Vav3 alters the reactivity state of cultured astrocytes. Here, a previously published study could already show that a treatment of primary astrocytes with metallothionein induced a reactive state of astrocytes with a significantly reduced activity level of RhoA which, however, had beneficial effects for the neurite outgrowth of neurons (Leung et al., 2010). Another hint for an altered reactive state of *Vav3*^–/–^ astrocytes is the enhanced wound closure observed in the scratch assay of this study (Figures 6A–H). Here, *Vav3*^–/–^ astrocytes showed a faster closure of the scratch area in comparison to wildtype astrocytes. Interestingly, it has been shown that a conditional knockout of Stat3 induced a higher activity level of RhoA which consequently led to a reduced migration of cultured astrocytes (Renault-Mihara and Okano, 2017; Renault-Mihara et al., 2017). The authors of this publication argue that the raised RhoA activity increases the actomyosin tone and reduces focal adhesion dynamics leading to a reduced migration speed of cultured astrocytes (Renault-Mihara and Okano, 2017; Renault-Mihara et al., 2017). Similar observations were made when Thy-1 receptor interactions were prolonged and induced an inhibition of RhoA, FAK, PI3K and Rac1 was activated and led to a higher migration behavior of astrocytes (Kong et al., 2013). A lowered RhoA activity could therefore reduce the actomyosin tone and increase focal adhesions inducing a higher migration speed, as observed in the scratch wound healing assay. The alterations of RhoA and of the cytoskeleton might furthermore induce the secretion of an altered chemokine/cytokine pattern.

## Conclusion

In conclusion, we could demonstrate as the first that a lack of the guanine nucleotide exchange factor Vav3 in astrocytes changes the pattern of released cytokines, chemokines and neurotrophic factors and enhance the outgrowth as well as the branching of dendrites in primary hippocampal neurons. The altered release of astrocytic factors is accompanied by a higher migration speed in a scratch wound healing assay. This observations might be used in order develop therapeutical strategies which target the astrocytic Vav3 and induce beneficial effects for neuroprotective or regenerative mechanisms in the CNS.

## Data Availability Statement

The raw data supporting the conclusions of this article will be made available by the authors, without undue reservation.

## Ethics Statement

The animal study was reviewed and approved by the Landesamt für Umweltschutz, Naturschutz und Verbraucherschutz, North Rhine-Westphalia, D-45659 Recklinghausen, Germany.

## Author Contributions

AF designed the study and supervised the study. JZ and DW performed the experiments and quantified as well as interpreted the data. DW drafted the manuscript. AF and JZ revised the manuscript. All authors contributed to the article and approved the submitted version.

## Conflict of Interest

The authors declare that the research was conducted in the absence of any commercial or financial relationships that could be construed as a potential conflict of interest.

## Publisher’s Note

All claims expressed in this article are solely those of the authors and do not necessarily represent those of their affiliated organizations, or those of the publisher, the editors and the reviewers. Any product that may be evaluated in this article, or claim that may be made by its manufacturer, is not guaranteed or endorsed by the publisher.

## Supplementary Material

Supplementary Figure 1 **(A)** ELISA standard curve for the determination of NT3 in the supernatant of wildtype and *Vav3*^–/–^ astrocytes after a conditioning time of 24 h. **(B)** The mean OD_450_ values of the analyzed wildtype and knockout probes revealed exceedingly low levels of NT3 in the supernatants which, furthermore, did not differ between both analyzed conditions.
