# Supplementary figures and images for: Vav3-Deficient Astrocytes Enhance the Dendritic Development of Hippocampal Neurons in an Indirect Co-culture System
Source: Front Cell Neurosci. 2022 Feb 14;15:817277. doi: 10.3389/fncel.2021.817277 (PMC8882586; doi:10.3389/fncel.2021.817277)

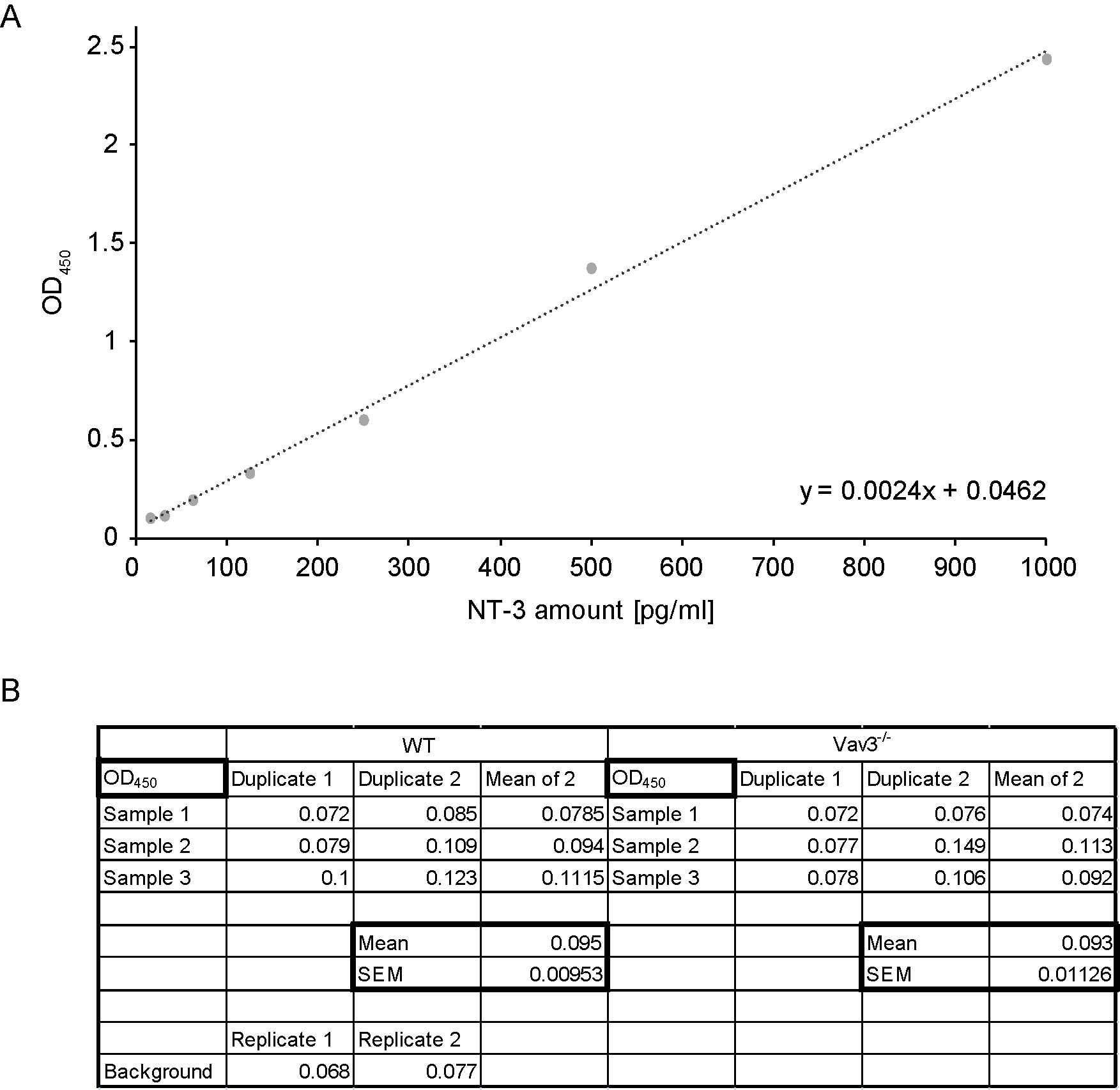

Supplement: Supplementary Figure 1 — (A) ELISA standard curve for the determination of NT3 in the supernatant of wildtype and Vav3–/– astrocytes after a conditioning time of 24 h. (B) The mean OD450 values of the analyzed wildtype and knockout probes revealed exceedingly low levels of NT3 in the supernatants which, furthermore, did not differ between both analyzed conditions. [file Image_1.TIF]
